# Transient remission of hyperparathyroidism after fine-needle aspiration biopsy

**DOI:** 10.1530/EO-22-0060

**Published:** 2022-08-31

**Authors:** Ana Rita Elvas, Andreia Martins Fernandes, Sara Reis, Joana Couto, Raquel G Martins, Jacinta Santos, Teresa Martins, Bernardo Marques, Joana Guimarães, Fernando J C Rodrigues

**Affiliations:** 1 Endocrinology Department, Portuguese Institute of Oncology of Coimbra FG, Coimbra, Portugal; 2 Pathology Department, Portuguese Institute of Oncology of Coimbra FG, Coimbra, Portugal; 3 Endocrinology Department, Egas Moniz Hospital, West Lisbon Hospital Center E.P.E., Lisboa, Portugal; 4 Endocrinology Department, Baixo Vouga Hospital Center E.P.E., Aveiro, Portugal

## Abstract

Primary hyperparathyroidism (PHPT) is the unregulated overproduction of parathyroid hormone (PTH), resulting in abnormal calcium homeostasis. PHPT is most commonly caused by a single adenoma of the parathyroid gland, which can have an intrathyroid location in rare cases. The measurement of intact PTH in the washout fluid obtained by ultrasound (US)-guided fineneedle aspiration (FNA) can be useful in clarifying the aetiology of these lesions. This study presented a 48-year-old man with a background history of symptomatic renal stone disease who was diagnosed with PHPT and referred to our Endocrinology department. A neck US revealed a thyroid nodule with a size of 21 mm in the right lobe. The patient underwent US-guided FNA of the lesion. The measurement of PTH in the washout fluid was significantly elevated. Following the procedure, he reported neck pain and noticed distal paraesthesias in the upper limbs. Blood test results showed significant hypocalcaemia and supplementation with calcium and calcitriol was started. The patient was closely monitored. Recurrence of hypercalcaemia was later observed, and the patient was submitted to surgery. We present a case of FNAinduced transitory remission of PHPT in a patient with an intrathyroid parathyroid adenoma. We conjecture that intra-nodular haemorrhage might have occurred, which temporarily affected the viability of the autonomous parathyroid tissue. A few similar cases of spontaneous or induced remission of PHPT after FNA have been previously described in the literature. This remission can be transitory or permanent, depending on the degree of cellular damage thus follow-up of these patients is recommended.

## Learning points

- Hyperfunctioning parathyroid lesions are susceptible to fine-needle aspiration (FNA)-induced damage.
- FNA biopsy is not generally recommended for the evaluation of primary hyperparathyroidism (PHPT), although it might be useful in selected cases, particularly by measuring parathyroid hormone in the washout.
- Considering the high recurrence rate, close follow-up of patients with FNA-induced remission of PHPT is recommended.

## Background

Primary hyperparathyroidism (PHPT) is a common endocrine disorder characterized by hypercalcaemia due to an unregulated overproduction of parathyroid hormone (PTH). PHPT is most commonly caused by a single adenoma of a parathyroid gland but can be caused by PHPT-related multiglandular disease in 10–15% of the cases. Parathyroid carcinoma is rare and occurs in less than 1% of the cases (Dandurand *et al.* 2021). Parathyroid glands are in close anatomic relationship with the thyroid gland, usually a total of four, though variation in number can be found. Ectopy of one or more glands is relatively common. In rare cases, they can have an intrathyroid location. The imaging techniques most frequently used to detect and locate abnormal parathyroids are ^99m^Tc-sestamibi scintigraphy and neck ultrasound (US). US is largely used in these patients as it is widely available, does not involve ionizing radiation and has a high sensitivity to detect these lesions (Bilezikian *et al.* 2014). In the case of an ectopic gland, especially intrathyroidal parathyroid adenoma, an abnormal gland may be considered as a thyroid nodule. Additionally, cytological differentiation between them is not easy due to overlapping features. Although not widely accepted due to the risk of parathyromatosis, the measurement of PTH in the washout fluid obtained by fine-needle aspiration (FNA) can be used in selected cases for clarifying the aetiology of these lesions (Suzuki *et al.* 2021). Surgery is the only curative therapy for primary hyperparathyroidism (PHPT). However, medical management with pharmacological agents is an option for some patients who have contraindications to surgery or are reluctant to undergo parathyroidectomy (Bilezikian *et al.* 2014).

## Case presentation

A 48-year-old man with complaints of fatigue and malaise was diagnosed with PHPT (total serum calcium concentration 12.4 mg/dL (reference range, 8.6–10.5 mg/dL) and PTH 462.1 pg/mL (reference range, 12–67 pg/mL) and referred to our Endocrinology department. He presented a background history of symptomatic renal stone disease and had already been submitted to lithotripsy.

## Investigation

**Figure 1:**
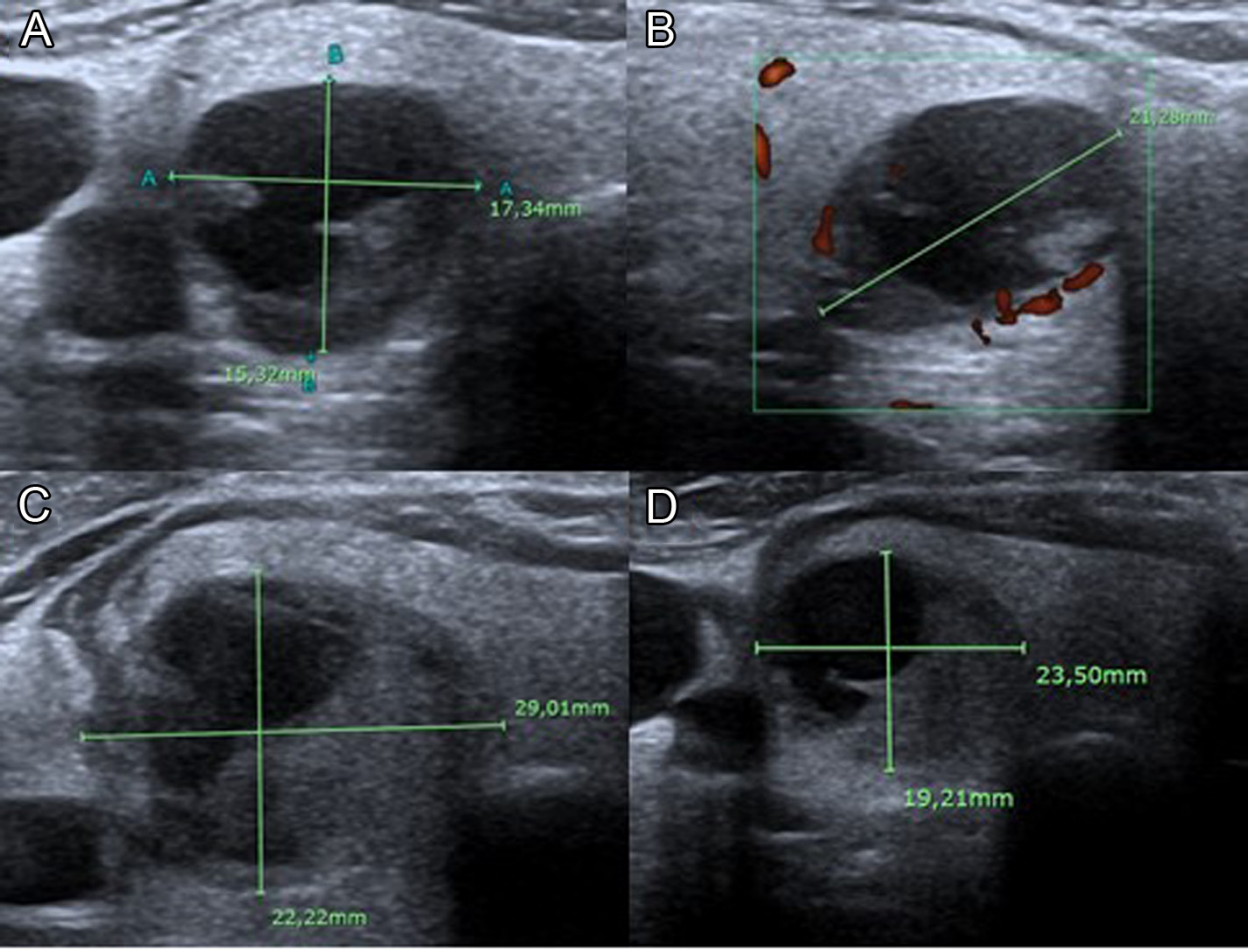
(A) Transverse and anteroposterior and (B) longitudinal diameters of the intrathyroid nodule in the ultrasound (US) images of the neck performed at the time of US-guided fine-needle aspiration (FNA). Image of the nodule 3 (C) and 10 days (D) after FNA.

A neck US was performed, and the only abnormal finding was a 21 mm predominantly cystic thyroid nodule in the right lobe (Fig. 1A and B). FNA using 25-gauge needle (two passes) with PTH washout to differentiate between an enlarged parathyroid and a thyroid nodule was undertaken. PTH measurement in FNA washout fluid was significantly elevated (PTH 7199 pg/mL).

**Figure 2:**
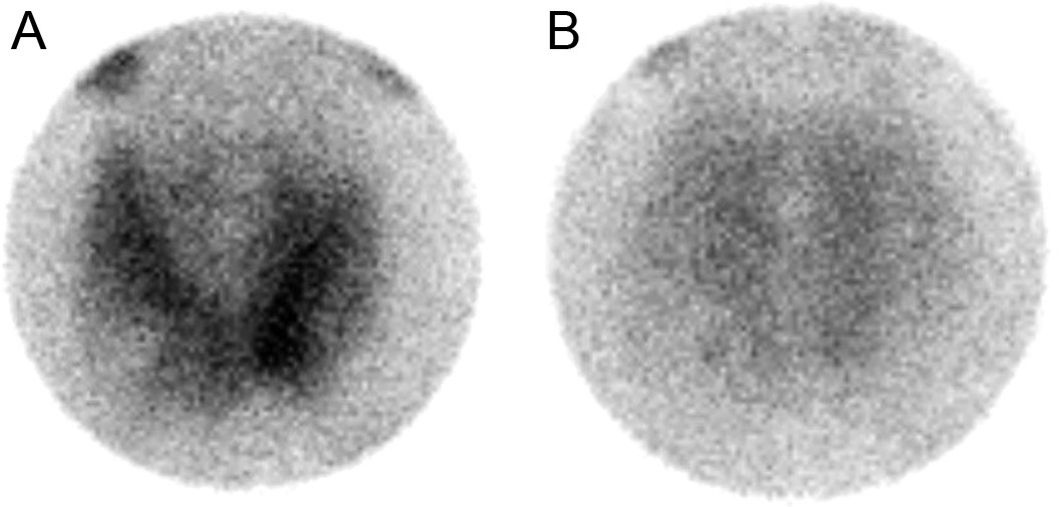
(A) Early and (B) delayed images of ^99m^Tc-sestamibi scintiscan.

Ten days after the procedure, he returned to our department reporting neck pain following FNA and has been noticing distal paraesthesias in the upper limbs. Blood test results showed hypocalcaemia (8.1 mg/dL) and reduction of PTH levels to 124 pg/mL. The patient reported symptomatic improvement after starting therapy with calcium plus vitamin D. Concurrently, a ^99m^Tc-sestamibi scan was performed and did not reveal any abnormalities suggestive of parathyroid disease (Fig. 2). Sequential US revealed an increase followed by a decrease in the nodule’s size from 29 to 23.5 mm in transverse diameter (Fig. 1: images C and D). The patient was kept under close monitoring. Forty-five days after FNA, recurrence of hypercalcaemia (11.5 mg/dL) was observed and the calcium plus vitamin D supplementation was discontinued. Elevated calcium levels persisted over time.

## Treatment

The patient was submitted to right thyroid lobectomy. During surgery, serum PTH decreased from 178.8 to 15 pg/mL within 10 min of the excision. The postoperative course was uneventful, with the exception of transient hypocalcaemia.

**Figure 3:**
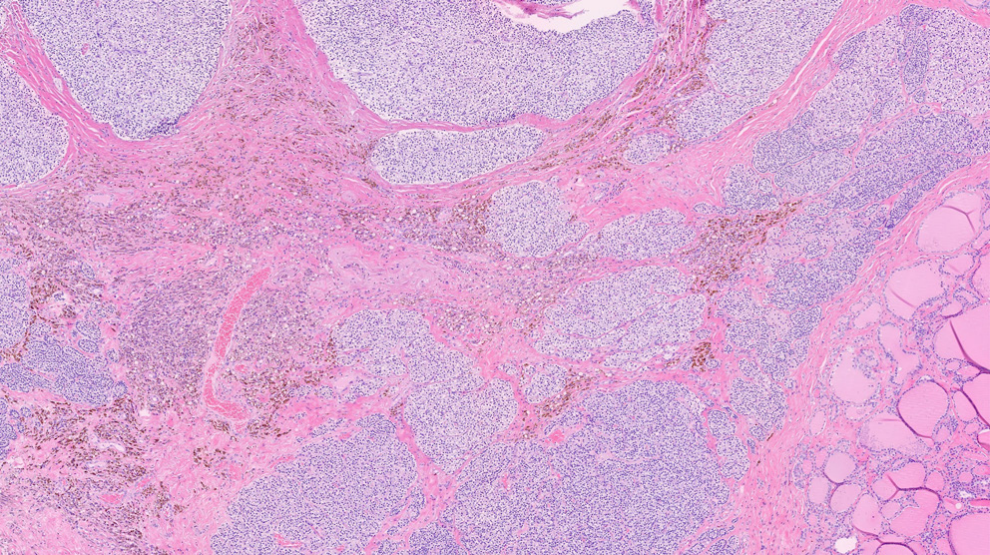
Intrathyroid parathyroid adenoma, with fibrosis and hemosiderin deposition.

Histopathology examination confirmed the presence of an intrathyroid parathyroid adenoma. The lesion had areas of fibrosis and hemosiderin deposition consistent with prior puncture (Fig. 3).

## Outcome and follow-up

Soon after surgery, calcium and PTH levels were within the normal reference range and have remained so ever since (last follow-up 17 months after surgery). Table 1 shows the biochemical profile changes after FNA and after surgery.

**Table 1:** The biochemical profile changes after FNA and after surgery.

Date	PTH, pg/mL^a^	Ionized calcium, mmol/L^b^	Total calcium, mg/dL^c^	Phosphate, mg/dL^d^	Time of follow-up
15-07-2019	462.1	1.55	12.4	2.0	Before US-guided FNA
19-07-2019	97.9	1.20	9.2	2.9	Three days after FNA
25-07-2019	124.0	1.09	8.3	3.3	Ten days after FNA
01-08-2019	107.6	1.16	9.5	3.6	During supplementation with calcium
29-08-2019	90.9	1.51	12	2.7	During supplementation with calcium
08-05-2020	173.1	1.49	11.6	2.9	Preoperative results
14-05-2020	15.5	1.14	8.8	2.3	First day after surgery
15-05-2020	38.2	1.1	8.7	3.6	Second day after surgery
02-11-2020	61	1.21	10.2	3.6	Six months after surgery
06-04-2021	104.7	1.16	9.9	3.6	Eleven months after surgery
12-10-2021	104	1.16	9.6	2.9	Seventeen months after surgery

^a^Reference range for intact parathyroid hormone, 18–80 pg/mL; ^b^Reference range for serum calcium, 8.6–10.5 mg/dL; ^c^Reference range for ionized calcium, 1.14–1.29 ng/mL; ^d^Reference range for phosphate, 2.5–5 mg/dL.

## Discussion

We present a case of FNA-induced transitory remission of PHPT in a patient with an intrathyroid hyperfunctioning parathyroid adenoma. Serum PTH and calcium dropped from 462.1 to 124 pg/mL and 12.0 to 8.1 mg/dL, respectively, following FNA. These unexpected changes along with neck pain suggest that FNA-induced haemorrhage led to remission of PHPT, which temporarily has affected the viability of the autonomous parathyroid tissue. Remarkably, the PTH level gradually began to rise 1 month after the procedure, demonstrating the damage was only temporary. In our case, a ^99m^Tc-sestamibi scan was performed during remission of PHPT, which may explain the negative result.

Spontaneous remission of PHPT due to nontraumatic necrosis, haemorrhage and infarction of a parathyroid adenoma is extremely uncommon, but it is a previously well-documented event, usually denominated as ‘parathyroid autoinfarction’, ‘autoparathyroidectomy’ or ‘parathyroid apoplexy’. This rare incident may range in presentation from asymptomatic to life threatening, with signs and symptoms of massive cervical or mediastinal haemorrhage, a condition requiring emergency neck exploration surgery. Most of the reported cases presented with acute hypocalcaemia, resulting from the adenoma’s necrosis and ineffectiveness of the remaining parathyroid glands to produce PTH, followed by a period of normocalcaemia at first and finally recurrence of the disease. Surgical treatment was performed in most cases, with only a few cases reported in which regular follow-up was initially chosen. This indicates that the apparent cure that follows necrosis of a parathyroid adenoma is potentially temporary, possibly explained by the presence of non-ischaemic adenomatous tissue having a potential to grow, resulting in the recurrence of hypercalcaemia at some stage. Therefore, long-term clinical and biochemical surveillance is advised (Novodvorsky *et al.* 2019).

In our case, remission of PHPT was caused by FNA of the parathyroid adenoma. This phenomenon is exceptional, and to the best of our knowledge, only six cases have been published so far (Ing & Pelliteri 2008, Maxwell *et al.* 2011, Kara *et al.* 2017, Falcetta *et al.* 2021, Ho *et al.* 2021). The cause remains unclear but has been suggested to be related to autoinfarction (necrosis without haemorrhage) or acute haemorrhage of the lesion after FNA, which can lead to an acute and dramatic reduction of calcium and PTH levels (Maxwell *et al.* 2011), while in some cases, the decrease is much less noticeable and entirely asymptomatic (Ing & Pelliteri 2008).

This remission can be transitory or permanent, depending on the degree of cellular damage. The patients reported by Kara *et al.* (2017) and more recently by Falcetta *et al.* (2021) experienced a long-term remission in 9-year and 1-year follow-up, respectively. In Ing & Pelliteri (2008), the complete aspiration of cystic fluid resolved hypercalcaemia for at least 16 months of post-FNA follow-up.

FNA is usually not recommended for parathyroid suspected tumours due to the chances of serious complications, such as massive haematoma, parathyromatosis and misdiagnosis as malignancy during pathological diagnosis. However, when the localization is unusual or parathyroid adenoma is mistaken for a thyroid nodule, FNA may be indicated or performed erroneously. Although cytomorphology alone may help distinguishing between parathyroid and thyroid lesions, it has remained a great challenge due to overlapping features (Suzuki *et al.* 2021). Some studies have reported that FNA with PTH measurement is helpful in diagnosing parathyroid disease (Ho *et al.* 2021). The performance of PTH washout only without cytology could be considered. However, this technique is mostly restricted to reoperative patients. In a study conducted by the Mayo Clinic Rochester, parathyroid FNA procedures were performed in 75 of 2265 parathyroid surgeries. For patients with PHPT referred because of difficulties with preoperative localization of their parathyroid adenoma, parathyroid FNA with PTH washout had a superior performance in comparison with parathyroid scanning or ultrasonography alone, exhibiting a positive predictive value of 100%, a sensitivity of 84%, a specificity of 100% and an accuracy of 84% (Bancos *et al.* 2012). However, further studies are needed to confirm the risk or justification of FNA for parathyroid adenomas and its importance in routine parathyroid localization.

In conclusion, our case, together with a literature review, suggests that hyperfunctioning parathyroid lesions are susceptible to FNA-induced damage. This procedure is not generally recommended for the evaluation of PHPT, although it might be useful in selected cases. Close follow-up of patients with FNA-induced remission of PHPT is recommended, considering the high recurrence rate.

## Declaration of interest

The authors declare that there is no conflict of interest that could be perceived as prejudicing the impartiality of this case report.

## Funding

This work did not receive any specific grant from any funding agency in the public, commercial or not-for-profit sector.

## Patient consent

Written informed consent for publication of their clinical details and clinical images was obtained from the patient.

## Author contribution statement

A R E drafted the manuscript. J C and F J C R were involved in critical revision of all drafts of the manuscript. J C, B M, R C M, J S, T M and J G were involved in patient care. All authors have approved the final version of the manuscript.
